# Features of alcohol advertisements across five urban slums in Kampala, Uganda: pilot testing a container-based approach

**DOI:** 10.1186/s12889-022-13350-2

**Published:** 2022-05-09

**Authors:** Monica H. Swahn, Jane B. Palmier, Alicia May, Dajun Dai, Sarah Braunstein, Rogers Kasirye

**Affiliations:** 1 Department of Epidemiology and Biostatistics, School of Public Health, Georgia State University, P.O. Box 3995, Atlanta, GA 30302-3995 USA; 2 Department of Health Promotion and Physical Education, Wellstar College of Health and Human Services, Kennesaw State University, Kennesaw, GA 30144 USA; 3 School of Public Health, University of Georgia, Athens, GA USA; 4 Department of Geosciences, Georgia State University, P.O. Box 4105, Atlanta, GA 30302 USA; 5 Uganda Youth Developmental Link, P.O. Box 12659, Kampala, Uganda

**Keywords:** Environmental scan, Alcohol, Alcohol marketing, Kampala, Uganda, Health, Global positioning system

## Abstract

**Background:**

Despite the high prevalence of alcohol use and marketing in many settings across sub-Saharan Africa, few studies have systematically sought to assess alcohol marketing exposure, particularly in vulnerable areas such as urban slums where alcohol is often highly prevalent but where educational programs and alcohol prevention messages are scarce.

**Objective:**

To pilot test the development and implementation of environmental scans of alcohol advertisements in five urban slums across different areas of Kampala, Uganda: Bwaise, Kamwokya, Makindye, Nakulabye, and Nateete.

**Methods:**

Each of the five scans was conducted in geographical circles, within a 500-m radius of a Uganda Youth Development Link (UYDEL) drop-in Center using a container-based approach. Using a Garmin GPS with photo capabilities and a tablet for data entry, teams of at least two trained researchers walked the main roads within the target area and gathered information about each alcohol advertisement including its location, type, size, and placement and other characteristics. Data with the GPS coordinates, photos and descriptive details of the adverts were merged for analyses.

**Results:**

A total of 235 alcohol adverts were found across all five data collection sites reflecting 32 different brands. The majority of the adverts (85.8%) were smaller and medium sizes placed by restaurants and bars, stores and kiosks, and liquor stores. The most frequently noted types of alcohol in the adverts were spirits (50.6%) and beer (30.6%).

**Recommendations:**

The pilot test of the methodology we developed indicated that implementation was feasible, although challenges were noted. Since monitoring alcohol marketing is key for addressing underage alcohol use and harm, the advantages and disadvantages of the approach we developed are discussed. Future research needs to strengthen and simplify strategies for monitoring alcohol marketing in low-resource settings such as urban slums which have unique features that need to be considered. Meanwhile, the findings may yield valuable information for stakeholders and to guide intervention developments and alcohol marketing policy to protect youth.

Despite the growing public health concerns about alcohol and related harm, alcohol remains one of the most commonly used substances globally, and contributes to about 5% of deaths and 5% of the global disease burden [1]. Research has shown that alcohol use is associated with harms including addiction and disorders [2, 3], other drug use [4], unintentional injuries [5, 6], physical fighting [7], criminal activity [4], suicidal ideation and attempts [8–10], and increased risk of HIV [11, 12]. In order to address this global public health issue, the World Health Organization (WHO) has prioritized the global reduction of the harmful use of alcohol with a particular focus on monitoring and technical support [13]. Even with limited data, it is still evident that low-income and middle-income countries, and particularly the vulnerable populations within these countries, bear an increased burden of disease and injury due to increasing alcohol consumption and limited or non-existent public health and prevention policies and programs [1]. In sub-Saharan Africa, specifically, alcohol use has been found to be associated with problem drinking [14], risky unprotected sex [15–18], mental illness [19–21] and road traffic accidents and injuries [22–24] among other health concerns and factors [25]. However, research on alcohol and harm across Africa lags behind other continents.

Alcohol use is affected by a range of individual and environmental factors as well as attitudes toward alcohol use, perceived susceptibility of alcohol use, peer drinking, accessibility of alcohol, and exposure to alcohol marketing as well as ownership of alcohol promotional items [26–31]. An empirical review of research in sub-Saharan Africa also demonstrated that alcohol use and risky sexual behaviors are linked to drinking venues and alcohol serving establishments [16]. However, systematic research on alcohol marketing exposure specifically remains relatively scarce across sub-Saharan Africa, although there is increasing interest in the topic [32–38]. Additionally, because of the strong alcohol industry presence and aggressive alcohol marketing in sub-Saharan Africa, there is grave concern about escalating alcohol use and harm [39, 40]. More specifically, research has outlined unethical alcohol advertisement and distribution practices in sub-Saharan Africa including giving underage youth free alcohol to consume [31, 32]. Additionally, researchers have also noted a high prevalence of vulnerable youth owning alcohol-branded products [31, 32] which underscore the reach of alcohol marketing strategies even among children and adolescents living in poverty.

Major barriers to progress in terms of understanding the prevalence, scope, and impact of alcohol marketing exposure in low resource settings are the relatively limited and complex tools and strategies used for assessing alcohol marketing density. In the “Guide for Measuring Alcohol Outlet Density” [41] the three most commonly approaches used (i.e., container-based measures, distance-based measures, and spatial access-based measures) are outlined [41]. Additionally, the specific advantages and disadvantages of each approach are described and compared. [41]. However, in our review of the published literature, the container-based approach is the only method used so far in establishing alcohol marketing density in sub-Saharan Africa and that approach was recently implemented in Tanzania [33] and in South Africa [36]. The container-based approach is also a recommended strategy for monitoring alcohol marketing by NGOs [42].

The purpose of this study was to determine the feasibility of implementing a container-based environmental scan assessing alcohol marketing in urban slums in Kampala. Moreover, we also wanted to describe the placement, features and content of the alcohol marketing. The project was designed to build on previous research and to inform the design of structural interventions such as alcohol counter marketing campaigns, to reduce harmful alcohol use in vulnerable communities. The need for structural interventions is particularly urgent for youth living in the slums of Kampala where educational programs and alcohol prevention messages are scarce and where the enforcement of the minimum legal drinking age of 18 years is rare. Our previous research of urban youth in Kampala outline grave health disparities and unmet needs, many of which stem directly from, or are exacerbated by, alcohol use [21, 43–51]. To our knowledge, a systematic environmental scan of alcohol marketing has never been conducted in this setting.

## Methodology development

The environmental scan protocol used in this project was modeled after previous research in alcohol outlet and marketing density typically described as a container-based approach [41]. Container-based measures of alcohol density are calculated based on the number of alcohol outlets in a specified geographic area (container). Our protocol was adapted using this approach to define the container area where the measurement of alcohol adverts would be performed and then assess alcohol marketing density within using a count of adverts, in addition to type, size, content and characteristics.

**Fig. 1:**
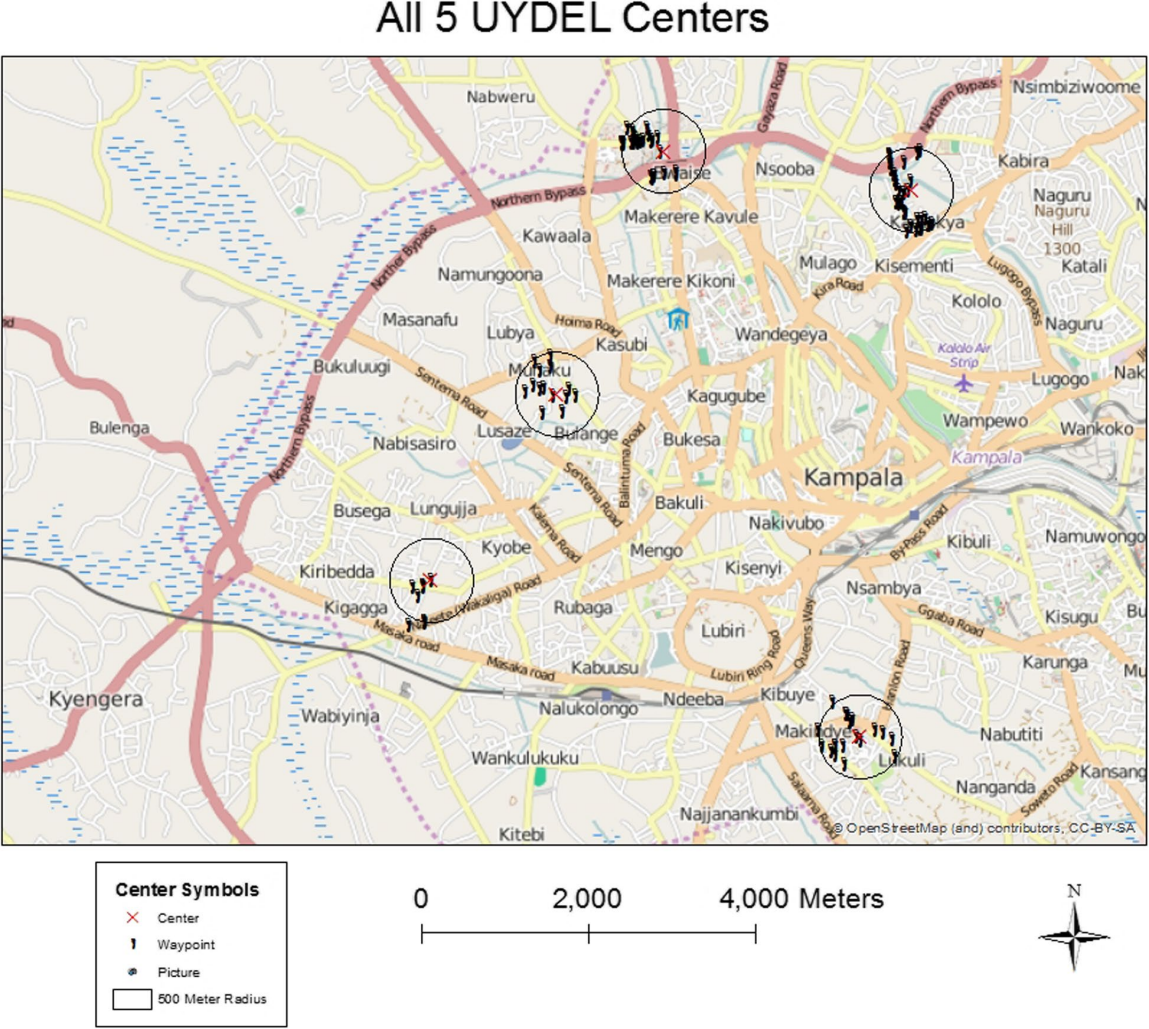
Centers, waypoints, and advertisements for all UYDEL centers

The research teams followed our newly developed protocol and used a data collection instrument (Qualtrics survey design software on Android tablets) to document the type of advertisements and the types of alcohol products advertised. Researchers (primarily graduate student research assistants), in teams of two people, visited the five targeted locations across Kampala slums within Bwaise, Kamwokya, Makindye, Nakulabye and Nateete (Fig. 1) in spring and summer of 2014. The target locations for each of the scans were the Uganda Youth Development Link (UYDEL) centers where most of our previous research has been located. UYDEL is an NGO that operates several drop-in centers for disadvantaged youth who live in the slums. The Centers offer youth prevention programs, vocational training and counseling services as well as referral for health screening and testing.

This project was conducted as part of a larger, multi-component study funded by the National Institutes of Health to assess alcohol marketing among vulnerable youth. In addition to the alcohol marketing assessment, it included a youth survey, youth focus groups, an NGO leader survey and focus groups, to inform the conceptual development of a structural intervention to reduce alcohol use, specifically by youth who reside in the slums. Given the aforementioned ongoing research studies, including surveys of the youth that UYDEL serves, the research team decided that the UYDEL drop-in centers were ideally suited as the base for the environmental scans of alcohol marketing given the research team’s familiarity with the community. However, for the purpose of testing the feasibility of the approach used, we could have selected other settings.

The environmental scan used Geographic Information System methods. The 500 M radius around each UYDEL Center was created using ESRI ArcGIS 10.1® and Google Earth®. We considered several different radii but settled on 500 M as it covered enough of a geographic area to give us enough data, but also because data collection could be completed in about half a day. The environmental scans were conducted on foot, with the teams walking in either direction for 500 m from the Center. Detailed street maps with a 500-m radius clearly demarcated around each study center site were created using ArcGIS ArcMap 10.1. The map used open source mapping of the Kampala area. This open-source map was created to ensure the teams covered all streets within the 500-m radius around the UYDEL centers. The teams systematically surveyed each tract once, first following the perimeter of each tract and then going through each street in the tract making sure to stay within a 500-m radius for each of the five centers. The Garmins GPS (described below) which had a proximity alarm were instrumental in providing the data collection team with the perimeter of the geographical area to be examined. Without that specific device and the alarm, it would have been very challenging for the team to stay within the boundary of the data collection areas as few other markers would have been available to identify the perimeter of the area to be scanned.

At each alcohol advertisement, a waypoint was created, and the content of the alcohol advertisements were logged by the data collection team. The researchers recorded each outdoor advertisement’s latitude and longitude using a GPS data logger and also took pictures and recorded locations (waypoints) of each of the outdoor advertisements with a Garmin GPS device. The Garmin devices that we used included a camera which facilitated linking photographs and waypoints. Additionally, the research team also filled out a data collection sheet on the tablet, providing additional information and context such as the type of advertisement, its location and content and whether there were any health warnings or restrictions pertaining to underage drinking for those less than 18 years of age (the legal drinking age is 18 years in Uganda).

The development of this approach required multiple field tests by several teams and repeat visits to the community to identify the different types of alcohol advertisements, their sizes and features. Because of the unexpected complexities noted in the field, we had to revisit the data collection sheets and field test them numerous times to ensure that relevant features were to be captured. Issues that were particularly complex were discussed by the entire team. An example of a complex issue was the collection of way points where there were multiple adverts, sometimes for the same alcohol product. Another issue that had to be addressed was the actual location of the advert. We decided not to count billboards that could be seen from within the data collection area but that were technically located outside the perimeter of the area to be scanned. We also had to consider if advertisements that had been ripped, torn or in other ways were perceived to be incomplete should be included. But, perhaps most unexpected in our methodology development were all the other highly prevalent marketing strategies and advertisements not typically seen in other settings which included branded signs, tablecloths, umbrellas, barware, coolers, painted exterior walls and other types. We opted not to include most of these as we were focused on traditional alcohol advertisements of specific alcohol products or sales outlets. However, we included painted advertisements that referenced a specific branded alcohol beverage.

Within the targeted area, teams photographed and recorded content of all alcohol advertisements visible from the main roads and paths only. Due to the irregularity of the streets and their exclusion from reliable geospatial sources, alleyways and paths not passable by cars or that were not indicated on the maps, were not included in the scan. In our field testing, the adverts not captured in those areas tended to be less frequent. Moreover, as we had decided to use the container-based approach with the ability to map the adverts, it seemed inconsistent to collect data in the geographical areas that could not be mapped because maps were not available.

Data regarding the advertisements, their content and waypoints (locations) were merged and aggregated for analyses. Also, the waypoints obtained from the Garmin GPS and the data loggers were imported into ArcMap.

### Equipment

#### Garmin GPS

Three Garmin Oregon 650 t® GPS devices were used as the navigation devices for the environmental scans. The Garmin Oregon 650 t is a touch screen device having the capacity to take waypoints (points that show the GPS coordinates of the advertisement) and photos of the advertisements. The Garmin also had a proximity alarm that was used to set the 500-m radius around each of the centers. This proximity alarm was a critically important feature for the data collection team to note the boundaries of the areas to be scanned. In order to conduct the scans, Garmin City Navigator® Eastern Africa NT maps were purchased and downloaded on each of the devices. The Garmin GPS devices were also color-coded to ensure that they matched with the data collected for the scan around each designated UYDEL center.

#### Data Loggers

Three Amod AGL3080® GPS Data Loggers were used as backup and confirmation for the Garmin GPS devices and to examine the specific path that the survey team walked during the scan at each center.

#### Nexus Android tablets

Google Nexus® Android Tablets with the Qualtrics® survey platform offline data collection application were used for the data collection. The tablets were also used to show the waypoints and pictures that were taken on the Garmin Oregon and to show the paths they walked throughout the data collection areas Finally, Google Earth was used to find the centers and create files that were used for making the maps.

### Measures

Descriptions of the advertisements were completed as part of a data collection sheet filled out on the Google Nexus tablets focusing specifically on the number of advertisements at each waypoint, whether permission was granted to take photos at each of the waypoints (if applicable), the type and placement of the advertisement, the type of establishment, the size of the advertisement, if a billboard was at the current waypoint or outside the container area, the alcohol type and brand, any kind of warning about underage/responsible drinking, description of advertisements and other comments the researchers wanted to note. Prior to implementation, the data collection tool was pilot tested multiple times and then modified to include specific brands and skip patterns to aid in the efficiency of the data collection. The skip patterns included the ability to identify a redundant advertisement at the same waypoint as captured by the Garmin. Permission to obtain photos was requested by the UYDEL staff member if there was a shopkeeper or proprietor present (none declined photographs of the alcohol adverts). For the purposes of this study, only printed and painted alcohol advertisements visible from the exterior were included in the data collection. However, we did not include other advertisement strategies or alcohol marketing such as branded barware, tablecloths, coolers and other similar products.

## Operationalization of the advertisement characteristics

The types of advertisements were classified as paper/flyer, poster, billboard, calendar, painted, banner, customized bar/restaurant, and other. Exterior placement was categorized as façade/exterior of building, free standing, store front, open store front door, and wall. The establishments were classified as store/kiosk, liquor store, retail shop, bar/restaurant, and other. The size categories of the advertisements were operationalized based on prior studies [52], and examination and measurement of adverts while field testing the methodology in Kampala, The sizes for the advertisements were categorized as follows: Paper/Flyer Size (< 20 X 30 cm or A4), small (< 30 X 70 cm), medium (< 40 X 120 cm), large (< 120 X 300 cm), X-Large (< 12sqm), XX-Large (< 18sqm), humongous (> 18sqm), and billboard over the road. Prior to finalizing our data collection strategy, we had considered measuring the advertisements. However, after repeated and failed attempts at directly measuring the adverts or using a ruler or yard stick to extrapolate the exact measurements, we decided to develop the advertisement categories and sizes, deciding that feasibility was more important than precision.

## Results

From the environmental scan, there were a total of 105 waypoints for a complete total of 235 advertisements. The characteristics and features of the alcohol adverts are presented in Table 1. The most common types of alcohol adverts were in the forms of paper/flyer and posters, comprising 85.8% of all the adverts collected. Over half (55.6%) of the exterior placements of the alcohol adverts were located outside an open store front or on an open door followed by the façade/exterior of building (20.1%). The most common establishments to display alcohol advertising were bars/restaurant, followed by stores/kiosks, and liquor stores. As for the sizes of alcohol adverts collected, the majority (88%) were considered paper/flyers, small and medium (less than 40 cm X 120 cm). Additonally, adverts were noted for 32 different brands. The brands with the greatest number of adverts were Bell Beer (*n* = 16), followed by Uganda Waragi (*n* = 15), Superior Gin (*n* = 13), Haveon Vodka (*n* = 12), Rider Vodka (n = 12) and Club Beer (n = 12). About three-fourths (*n* = 173, 73.9%) of the advertisements had a warning about the harmful effects of alcohol and underage drinking.

**Table 1:** Characteristics and features of 235 alcohol advertisements obtained across 5 data collection sites in Kampala, Uganda

Advert Characteristics	Adverts (n)	
**Type of Advertisement**		Percent of Total Adverts (%)
Paper	119	50.8
Poster	82	35.0
Billboard	4	1.7
Calendar	1	0.4
Painted	7	3.0
Banner	6	2.6
Customized Bar/Restaurant	8	3.4
Other	8	3.0
Total	235	100
**Exterior Placement**		Percent of Total Adverts (%)
Façade/Exterior of Building	48	20.1
Free Standing	12	5.1
Store Front	44	18.8
Open Store/Front Door	130	55.6
Wall	1	0.4
Total	235	100
**Type of Establishments**		Percent of Total Adverts (%)
Store/Kiosk	54	23.1
Liquor Store	54	23.1
Retail Strip	11	4.7
Bar/Restaurant	73	31.2
Other	31	13.2
Total	223^a^	100
**Advert Size**		Percent of Total Adverts (%)
(< 20 X 30 cm) or A4	121	52.1
Small (< 30 X 70 cm)	52	22.2
Medium (< 40 X 120 cm)	32	13.7
Large (< 120 X 300 cm)	19	8.1
X-Large (<12sqm)	6	2.6
Humongous (>18sqm)	5	2.1
Total	235	100
**Types of Alcohol**		Percent of Total Adverts (%)
Beer	72	30.6
Wine	1	0.04
Spirits	119	50.6
Other	11	4.7
Multiple Types	32	13.6
Total	235	100

^a^Type of establishment were missing for 11 adverts

## Discussion

The purpose of this study was to determine the feasibility of implementing a container-based environmental scan assessing the features of alcohol adverstising and its content in urban slums in Kampala. Assessing the features and content of alcohol advertisements is important and can inform the design of structural interventions, such as alcohol counter marketing campaigns intended to reduce alcohol use and alcohol-related harm in vulnerable communities. Overall, we found that the implementation was feasible in this low resource setting. However, we did encounter some challenges worth noting in the implementation of a container-based approach for assessing alcohol advertisements in this setting. The project turned out to be much more labor- intensive than anticipated as it required multiple iterations of data collection to enable a clear and systematic approach that captured the somewhat unique alcohol advertisements in Kampala. It also required substantial expertise and resources in terms of mapping software, sophisticated and relatively expensive technology. Despite these challenges, the findings from this pilot test may inform and guide methodological advancements for assessing alcohol marketing exposure in urban, low-resource settings.

To our knowledge, this is the first environmental scan of alcohol marketing to use a geospatial container-based approach to examine alcohol marketing in urban slums in sub-Saharan Africa. While the topic of alcohol marketing exposure and its assessment have gained some interest by researchers relatively recently [31–37], few studies have yet to systematically assess alcohol marketing exposure. While we chose the container-based approach which has been used previously, those studies were in very different contexts in both Tanzania [33] and South Africa [36].

Our findings show that alcohol marketing is highly visible within the urban slums in Kampala. Overall, we found 235 adverts in 105 locations indicating that multiple adverts are typically clustered or placed together. Moreover, our findings show that the alcohol marketing in this setting is primarily comprised of smaller size adverts that specifically promote spirits and beers. These adverts are most often located as part of retail establishments such as bar and restaurants, liquor stores and other smaller stores and kiosks. These findings illustrate that alcohol marketing within these urban slums may look very different than in other settings such as the urban city centers, where the larger billboards may be the key features of alcohol industry marketing practices. The fact that most alcohol adverts were placed by retail establishments provides an obvious focal point for structural interventions that may seek to reduce or shield alcohol marketing exposure, and alcohol access, for children and underage youth.

Prior to our alcohol marketing scans, anecdotal information and preliminary research had indicated high levels of alcohol marketing and unethical marketing practices, some of which we have outlined in previous research [31, 32]. This environmental scan provided valuable data to guide the intervention development. In particular, the fact that advertisements were typically clustered together, provide clear target areas where alcohol counter marketing efforts can be implemented. Additionally, because most of the advertisements consisted of smaller posters placed by alcohol outlets, involving bar and shop owners as community stakeholders in initiatives to protect youth should be a key strategy for community prevention and structural interventions. In fact, we shared the findings from our pilot test with bar and restaurant owners and other community members during a stakeholder forum. In this forum we provided additional information of alcohol use and harm among youth in their communities while also presenting the content of the advertisements and its scope. In that context and with a focused discussion, the importance of addressing alcohol advertisement became more clear to stakeholders who may not previously have considered the role of marketing in underage alcohol use and related harm.

It is important to also note, as mentioned previously, that conducting this type of alcohol marketing assessment was labor- intensive and, involved the use of relatively expensive tools and technology that may not be readily available in low-resource settings. As such, we recommend the development of new tools and strategies specifically for use in these urban settings that can be led by NGOs and community-based organizations [42] to greatly simplify the ongoing monitoring of alcohol marketing to drive community changes and improvements. These new strategies and tools should consider the metrics to be used to allow for comparisons across settings. Basically, these strategies and tools should enable us to determine what level of alcohol marketing can be considered as high and the units by which it can be most effectively measured.

As with any pilot projects, we noted both strengths and limitations to our specific approach that will guide our future work. The strengths of our approach included the use of rigorous training of the field research team, building on previous research and use of the latest technology available at the time to capture both photographs and GPS coordinates of the location of the alcohol advertisements. We also validated the paths taken by the research team across the target areas using data loggers. Accordingly, the data is rich with details that can assist us in furthering the methodology and to also provide specific details about the extent and nature of alcohol marketing in these geographic areas for the purposes of implementing interventions. However, there are also important limitations of the protocol and its implementation that have also provided several important lessons learned. A major limitation was in the logistical details of data collection, data transfers and aggregation across data collection tools, specifically the Garmin GPS and Tablet data. Despite rigorous training, we noted different approaches by the data collection teams. Fortunately, many of these discrepancies in data collection could be addressed immediately using the photographs taken of all the advertisements. The most significant barrier to the project implementation was the limited map availability of the target areas. Because most paths in the target areas were not visible on the maps available, the teams did not conduct the scans in those areas. As such, the number of advertisements captured and described are likely a substantial undercount of the advertisements in the entire target areas. Moreover, the targeted areas around the UYDEL drop-in centers were arbitrarily selected for convenience and familiarity by project staff. As such, these areas may not be representative of the broader and growing slums across Kampala. However, the key goal was to develop the protocol and to assess the feasibility of its implementation and to guide the intervention developments. Another limitation is that the data is older, collected in 2014. However, given the scarcity of these environmental scans, the data and methods used will be a great resource to others contemplating similar work. Additionally, while our work in Uganda is ongoing, we have not noted any changes to alcohol marketing and its content in our more recent work in Kampala that would alter the conclusions presented in this paper.

Despite the limitations, this project demonstrates that it is feasible to conduct an environmental scan of alcohol advertisements in the slums of Kampala. The findings also show that alcohol marketing is widespread and that there are clear patterns that can be targeted for interventions as well as future alcohol marketing research. We suggest that further research incorporate our lessons learned to enhance data collection feasibility, accuracy and scope. This is a critically important area for research. Currently there are exciting new conceptual developments to guide the advancement of alcohol research in low-income countries [53] as well as a renewed call to action [54] regarding the WHO global strategy to reduce alcohol harm [13], particularly in low-resource settings where the overall alcohol burden is the highest.

## Data Availability

The data used and analysed during the current study are available from the corresponding author on reasonable request.
